# Under pressure

**DOI:** 10.7554/eLife.18435

**Published:** 2016-07-15

**Authors:** Ulrich Z Hammes

**Affiliations:** Department of Cell Biology and Plant Biochemistry, Regensburg University, Regensburg, Germanyulrich.hammes@biologie.uni-regensburg.de

**Keywords:** phloem, morning glory, sieve element, pressure flow hypothesis, long distance transport, Münch, Other

## Abstract

The movement of water by osmosis causes pressure differences that drive the transport of sugars over long distances in plants.

**Related research article** Knoblauch M, Knoblauch J, Mullendore DL, Savage JA, Babst BA, Beecher SD, Dodgen AC, Jensen KH, Holbrook NM. 2016. Testing the Münch hypothesis of long distance phloem transport in plants. *eLife* **5**:e15341. doi: 10.7554/eLife.15341

**Image** Tall plants have larger pores in structures called sieve plates than short plants

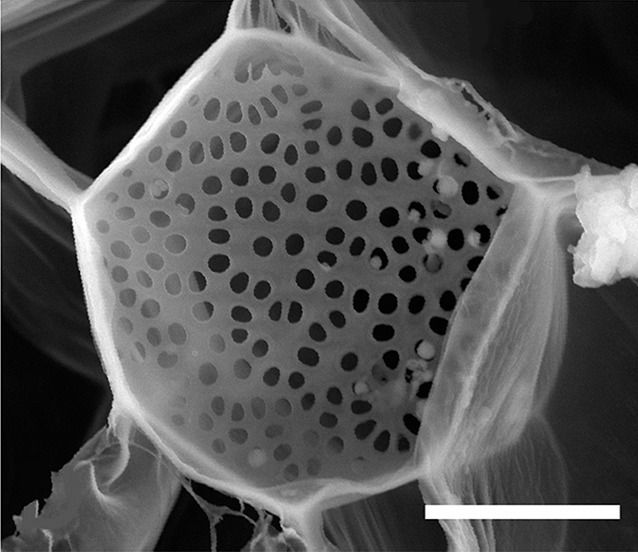

Plants use photosynthesis to make sugar in their leaves and other green tissues. They also move excess sugars from these “source” tissues to other parts of the plant, such as the seeds, fruits and roots. In addition to playing crucial roles in the life of the plant, these "sink" tissues are often the edible part of the plant. Indeed, virtually every calorie we consume has travelled from a source tissue to sink tissue at least once, so the system used to transport sugars in plants has a big influence on crop yields.

The long distance transport of sugars occurs in a tissue called phloem. In tall trees, source tissues and sink tissues can be separated by more than 100 meters: it would take over 300,000 years for sugars to diffuse over such a distance, so there must be other mechanisms in place. Now, in eLife, Michael Knoblauch of Washington State University and colleagues tackle long standing questions in the field of phloem transport (Knoblauch et al., 2016).

In flowering plants, cells called sieve elements form the tubes that transport sugars and other molecules through the phloem. These cells adopt a “zombie-like” state as they mature: although they are living cells, they are only able to carry out a limited number of processes compared to other plant cells. The sieve elements are separated by sieve plates, which contain large pores that allow fluid to flow through the phloem. Alongside the phloem, another type of tissue called the xylem transports water and ions away from the roots. Although the phloem and xylem play different roles in the plant, they also depend heavily on each other.

In 1930, the German botanist Ernst Münch proposed that pressure differences between the source and sink tissues are responsible for transport through the phloem (Münch, 1927). In the phloem of source tissues, sugars are highly concentrated, so water is drawn into the sieve tubes from the neighboring xylem. This increases the hydrostatic pressure, which is counteracted by the cell walls of sieve elements, leading to an increase in the turgor pressure. In sink tissues, on the other hand, sugars are consumed, which reduces their concentration in the phloem: this allows water to flow back into the xylem and leads to a decrease in the turgor pressure.

Münch proposed that the difference in turgor pressure between the source and the sink is sufficient to drive long-distance transport without the need of additional energy. This so-called “pressure flow hypothesis” is very intuitive and has been taught at universities for decades despite there being only limited experimental evidence to support it. It is technically challenging to perform experiments on phloem because the tissue is often deeply embedded in the plant and it is difficult to isolate sieve tubes.

The Knoblauch lab has a long history of developing techniques to measure the biophysical characteristics of flow through the phloem and xylem (Knoblauch and van Bel, 1998; Mullendore et al., 2010; Froelich et al., 2011; Jensen et al., 2012; Knoblauch et al., 2014). Now, they have used these methods to calculate the flow of fluid through the phloem of a type of vine called Morning Glory (*Ipomea nil*), which can grow up to 15 meters in length.

If the Münch hypothesis is right, there should be differences in turgor pressure and phloem conductivity (the ability of fluid to move through the phloem) between tall and short plants. Knoblauch and colleagues – who are based at Washington State University, Harvard University, the Brookhaven National Laboratory and the Technical University of Denmark – allowed individual morning glory plants to grow to several different lengths (Figure 1). The plants had their lower leaves removed so that the only leaves remaining were on the top four meters of each plant. The experiments demonstrate that taller plants have larger differences in turgor pressure between source and sink compared to shorter plants. Furthermore, phloem tubes have higher conductivities in taller plants than in shorter plants because the pores in their sieve plates are larger.

**Figure 1.**
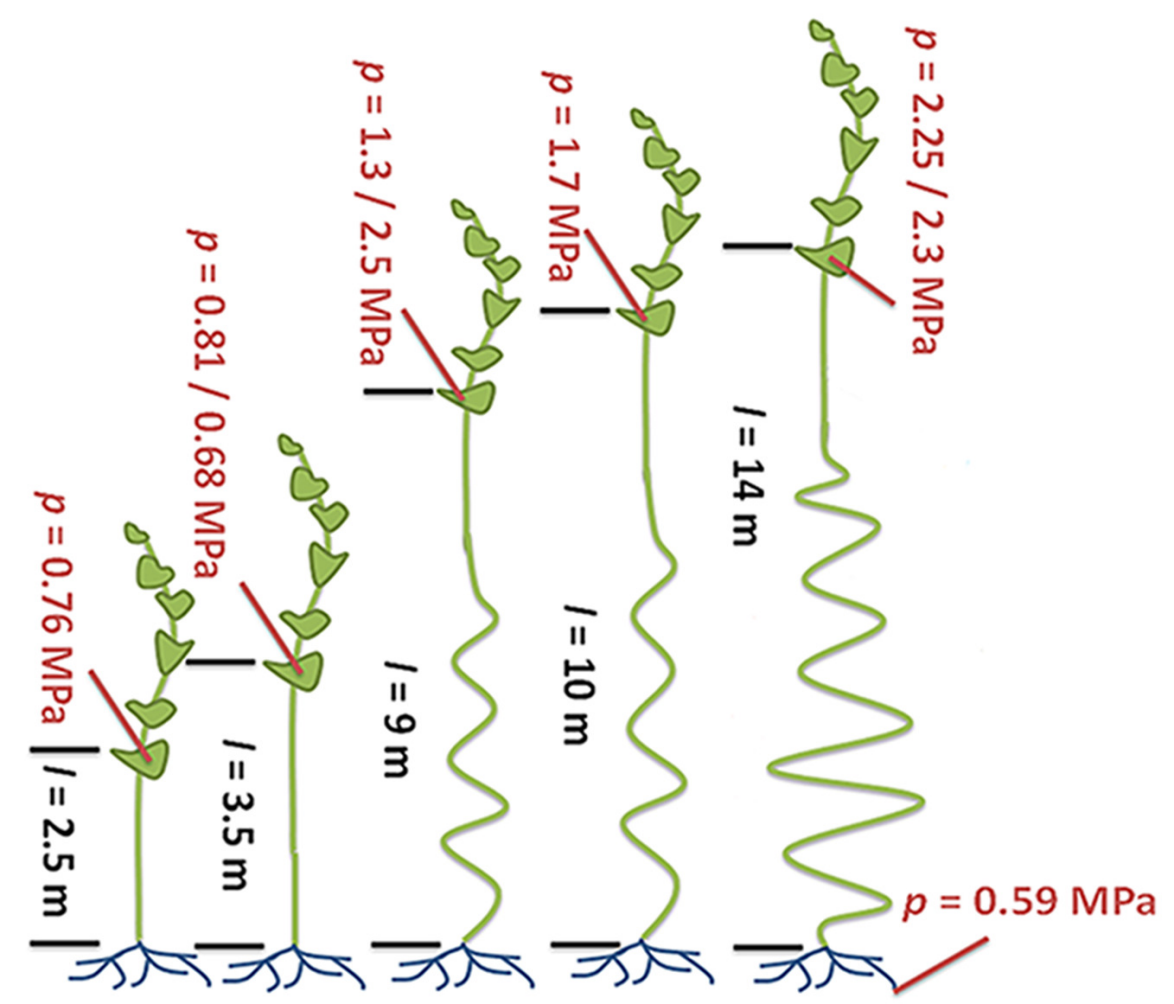
Turgor pressure drives long-distance transport through the phloem. Sugars produced in plant leaves (outlined in dark green) are transported to the roots (blue) or other “sink tissues” around the plant (not shown). Knoblauch et al. grew morning glory plants to different heights and removed the lower leaves so that the only leaves remaining were on the top four meters of each plant. All of the plants have similarly low turgor pressures in the root phloem. Plants with a short distance between the leaves and the roots (black text; distance (l) is given in meters) maintain relatively low turgor pressures (red text; pressure (p) is given in megapascals) in the phloem within the leaves. Taller plants maintain higher turgor pressures in their leaf phloem. The ability of fluid to flow through the phloem (conductivity) is also higher in the taller plants (not shown). Illustration adapted from Knoblauch et al. (2016).

This study provides a long-awaited experimental proof of Münch’s pressure flow hypothesis. One of the most striking findings is the observation that, in contrast to a generally accepted theory, the unloading of sugars from phloem in sink tissues is very likely to involve additional inputs of energy. This implies that transporter proteins that unload nutrients from the phloem – like the SWEET and UmamiT proteins – play much more important roles than previously thought (Chen et al., 2012; 2015; Muller et al., 2015). Their biophysical properties need to be investigated in much greater detail to understand their role in phloem transport. Another question for future research is how plants control the distribution of resources between tissues for storage or growth. Last but not least, the precise molecular mechanisms of phloem loading, particularly in trees, are still a matter of debate that needs to be investigated.
